# Theoretical and Experimental Analyses on the Sound Absorption Coefficient of Rice and Buckwheat Husks Based on Micro-CT Scan Data

**DOI:** 10.3390/ma16165671

**Published:** 2023-08-17

**Authors:** Shuichi Sakamoto, Kentaro Toda, Shotaro Seino, Kohta Hoshiyama, Takamasa Satoh

**Affiliations:** 1 Department of Engineering, Niigata University, Ikarashi 2-no-cho 8050, Nishi-ku, Niigata 950-2181, Japan; 2 Graduate School of Science and Technology, Niigata University, Ikarashi 2-no-cho 8050, Nishi-ku, Niigata 950-2181, Japan; f21b105g@gmail.com (K.T.); t17a057k@gmail.com (S.S.); kohtahoshiyama@gmail.com (K.H.); 3 Fukoku Co., Ltd., 6 Showa Chiyoda-machi, Oura-gun, Gunma 370-0723, Japan; takamasa_satoh@fukoku-rubber.co.jp

**Keywords:** rice husks, buckwheat husks, micro-CT scan, tortuosity, sound absorption coefficient

## Abstract

In this study, the sound absorption coefficients of rice and buckwheat husks were estimated. Computed tomography (CT) images were processed to determine the circumference and surface area of voids in the granular material, and the normal incident sound absorption coefficients were derived. In addition, the tortuosity, which expresses the complexity of the sound wave propagation through the structure, was measured for each material. The theoretical sound absorption coefficients were then compared to the measured sound absorption coefficients with and without consideration of the tortuosity. A correction factor was used to bring the surface area of the granular material closer to the actual surface area and observed that the tortuosity obtained theoretical values that matched the trend of the measured values. These results indicate that using CT images to estimate the sound absorption coefficient is a viable approach.

## 1. Introduction

Plant biomass from grains, vegetables, and trees is used for various purposes, including food, fuel, and building materials. The shape and size of these materials indicate that they may also have useful sound absorption effects. For example, rice straw has a microtubular structure [1,2,3]. Bastos et al. made sound-absorbing panels from coconut fiber [4], Gabriel et al. did the same with corn fiber [5], Khai et al. used oil palm fiber [6], and Sezgin used discarded tea leaves [7]. Umberto et al. made sound absorbers from pulverized cane [8], and Rubén et al. used cork bark [9]. Moreover, previous reports of biomass sound-absorbing materials can be found in various instances, such as natural bamboo fibers [10], straw [11], sugarcane wasted fibers [12], and Tatami mats [13]. These studies were all based on the principle that the boundary-layer viscosity of air at the walls of granular/flake-filled structures attenuates the energy of incident sound waves. The continuous voids of such structures cause them to exhibit the same acoustic behavior as porous materials, and the acoustic properties can be adjusted according to the layer thickness, grain size, and packing structure [14,15].

Japan generates about 1.6 million tons of rice husks [16] and 20,000–30,000 tons of buckwheat husks [17] annually, of which much is incinerated and disposed of without being utilized. Rice husks are a byproduct from the production of rice, which is a staple food of Japan and is widely dispersed throughout the country, so a stable supply can be expected. Buckwheat husks are commonly used to make pillows, but this application has been decreasing, so most husks are at present disposed of as waste [17]. Recently, restrictions have been placed on burning these materials in the open due to environmental concerns, which has made their disposal a serious problem. The sound absorption of rice and buckwheat husks has previously been reported [1], but the sound absorption coefficient was only measured. A method of estimating the sound absorption coefficient has not been developed. This is because the packing structures of rice and buckwheat husks do not have a periodic arrangement and vary with the grain size, which makes constructing a mathematical model difficult. However, rice and buckwheat husks maintain a stable flake shape after threshing and are expected to exhibit acoustic properties as granular materials with stable shape, such as porous materials. This study elaborates on the sound absorption principle caused by the viscous friction of the boundary layer of the wall surface, which has been discussed in a previous study [15]. The sound wave attenuation discussed in this paper is independent of microscale molecular structures. Meanwhile, nano-fibers, such as nanocellulose fibers, act to enhance the sound absorption properties of nonwoven fabrics [18].

In this study, the sound absorption coefficients of rice and buckwheat husks were estimated. Computed tomography (CT) images were processed to determine the circumference and surface area of voids in the granular material, and the normal incident sound absorption coefficients were derived. However, micro-CT scans have only been used to observe plant structure conventionally [19]. In addition, the tortuosity, which expresses the complexity of the sound wave propagation through the structure, was measured for each material. The theoretical sound absorption coefficients were then compared to the measured sound absorption coefficients with and without consideration of the tortuosity.

## 2. Experimental Measurements

### 2.1. Sound Absorption

**Figure 1:**
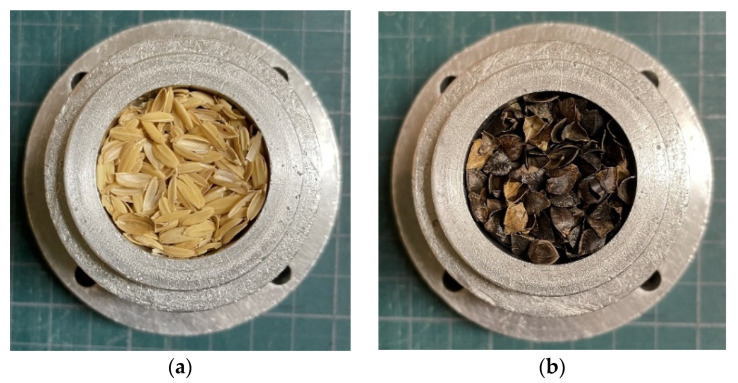
Samples: (**a**) rice husks; (**b**) buckwheat husks.

Two types of biomass materials were tested for their sound absorption: rice husks and buckwheat husks. Both materials were from Japan. Figure 1 shows the measurement samples, and Table 1 presents the specifications. Each sample was used to directly fill an aluminum alloy tube with an inner diameter of 29 mm and length of 20 mm.

During threshing, rice and buckwheat husks separate on their own owing to their flake shapes (Figure 1), and stable shapes can be obtained. This study focused on rice and buckwheat husks owing to their ability to form a stable shape through this process. Therefore, this study proposes to leverage these naturally occurring and readily obtainable threshed shapes. In general, rice and buckwheat husks do not attract insect damage after being washed and dried, as they retain minimal seed powder. Note that, if the rice husks were heated to ash, they could manifest the sound absorption properties of finer-grained powders [20].

**Figure 2:**
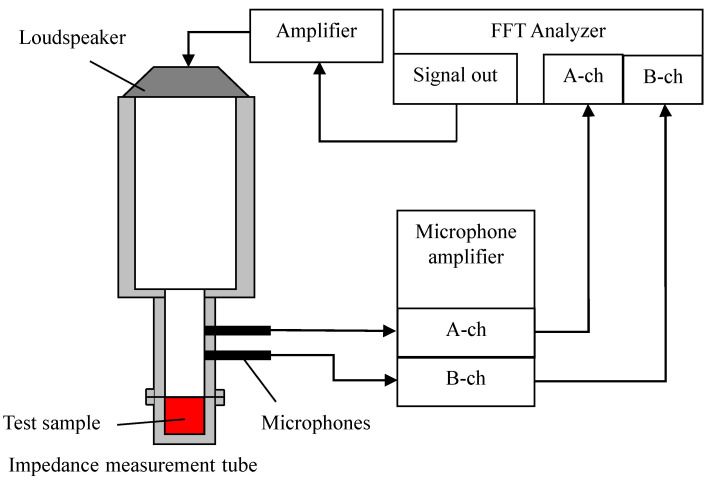
Configuration of the two-microphone impedance tube used for sound absorption measurements.

As shown in Figure 2, a Brüel and Kjær Type 4206 (Nærum, Denmark) two-microphone impedance measurement tube was used to measure the normal-incident sound absorption coefficient. A sound wave based on a sinusoidal signal from a signal generator with a built-in fast Fourier-transform (FFT) analyzer DS-3000 fabricated by Ono Sokki (Yokohama, Japan) was output into the measurement tube containing the sample. The sound pressure in the tube was measured by the two microphones, and the transfer function between the sound pressure signals was calculated by using the FFT analyzer. The measured transfer function was used to derive the normal incident sound absorption coefficient in accordance with ISO 10534-2 [21]. The critical frequency at which a plane wave forms depends on the inner diameter of the acoustic tube. Because the tube used in this study had an inner diameter of 29 mm, the upper limit of the measurement range was 6400 Hz. The voltage of the input signal to the loudspeaker was 0.2 V.

### 2.2. Tortuosity

The tortuosity is an acoustic parameter that expresses the complexity of the path of a sound wave passing through a sound-absorbing material with a complex internal geometry. In this study, ultrasonic sensors were used to measure the tortuosity of the materials so that its effect on sound absorption can be considered. The tortuosity *α*_∞_ can be derived from the velocity of sound in air *c*_0_ and the apparent velocity of sound in a material *c* [22]:

(1) $$\alpha_{\infty }={\left(\frac{c_{0}}{c}\right)}^{2}$$

If the sample material contains no obstacles and the sound wave travels in a straight path, then *α*_∞_ = 1 because the *c*_0_ = *c*. If the path is complex, then *c* decreases, and *α*_∞_ becomes >1. In other words, a greater tortuosity indicates a longer path for sound waves within a structure. This effect is similar to an increase in the material thickness.

**Figure 3:**
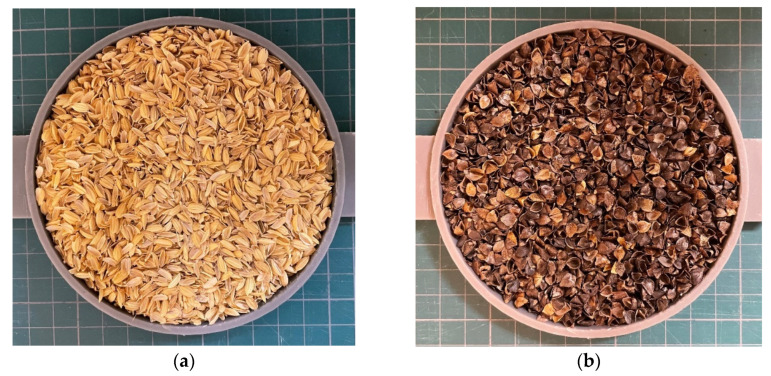
Samples for tortuosity measurement: (**a**) rice husks; (**b**) buckwheat husks.

**Figure 4:**
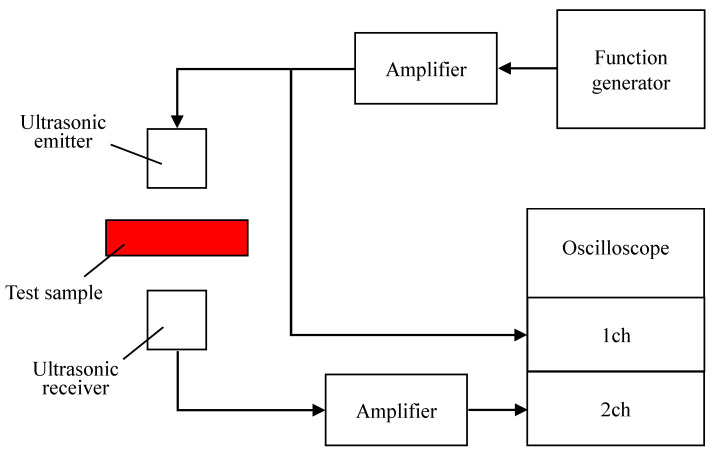
Tortuosity measurement setup.

To measure the tortuosity, sound waves were transmitted through the bottom of the sample holder, as shown in Figure 3. The wire mesh bottom allowed only sound waves to penetrate the sample, and no granular material fell. The sample holder was fabricated from light-cured resin using the 3D printer Form2 manufactured by Formlabs Inc. (Somerville, MA, USA). Figure 4 shows the tortuosity measurement setup.

**Figure 5:**
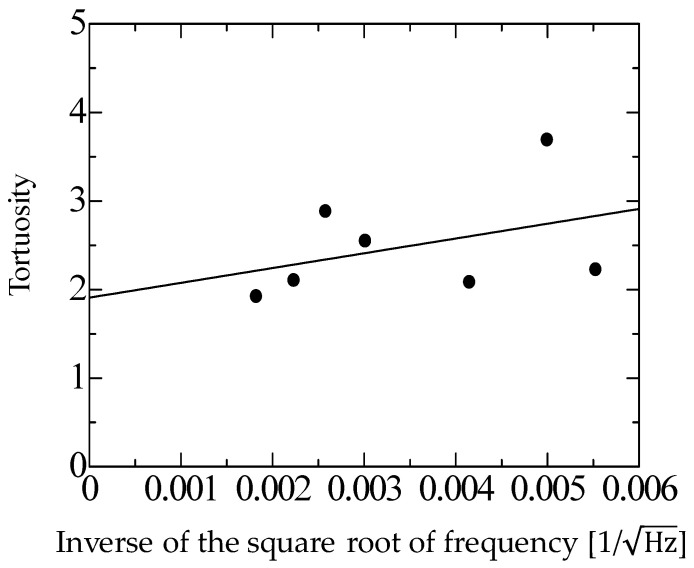
Measured tortuosity of the rice husks.

**Figure 6:**
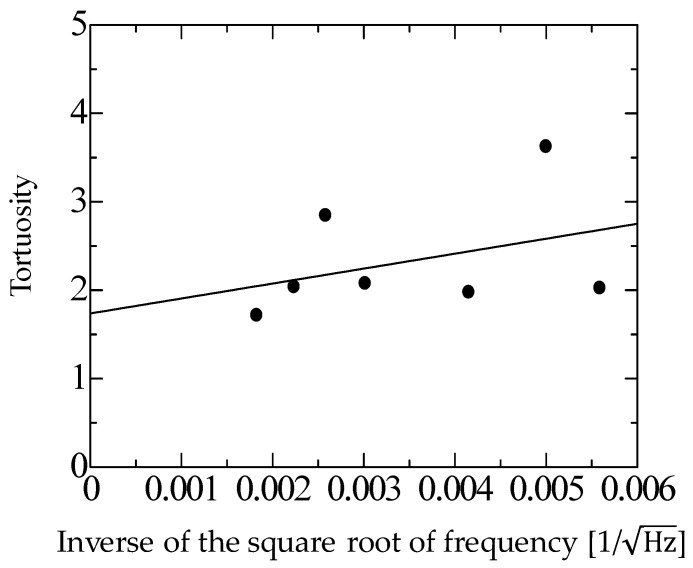
Measured tortuosity of the buckwheat husks.

The tortuosity was measured by the same method reported previously [14]. Ultrasonic sensors with central frequencies of 32.7, 40, 58, 110, 150, 200, and 300 kHz were used. First, the tortuosity *α*_∞_ was measured for each filling structure at each frequency. Then, the inverse of the square root of the measurement frequency was taken as the value on the horizontal axis, and the tortuosity *α*_∞_ at each frequency was plotted as the value on the vertical axis. The least-squares method was applied to obtain a linear approximation of these points, which yielded a straight line that increased steadily to the right. The tortuosity *α*_∞_ of the material was defined as the extreme value of the tortuosity when the frequency of the approximated line was set to infinity, i.e., the *y*-intercept of the graph. To improve the signal-to-noise ratio and measurement accuracy, the results of 300 measurements were summed synchronously. The signals were measured at a resolution of 16 bits. Figure 5 and Figure 6 show the measured tortuosities, which were *α*_∞_ = 1.92 for rice husks and *α*_∞_ = 1.74 for buckwheat husks.

## 3. Theoretical Analysis

### 3.1. Overview

**Figure 7:**
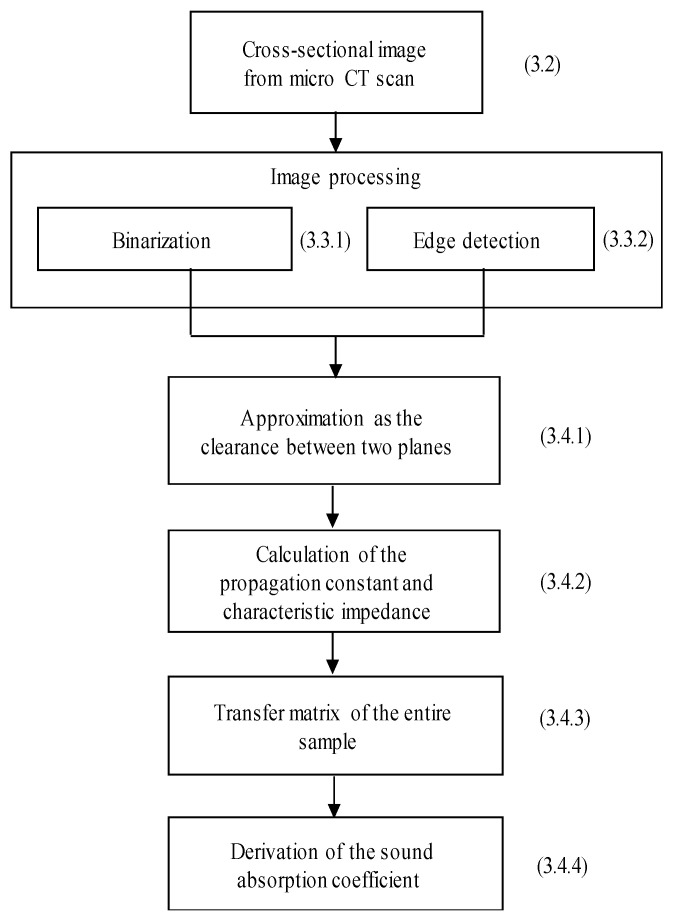
Overview of the theoretical analysis.

Figure 7 shows a flowchart of the theoretical analysis used to derive the sound absorption coefficient. Micro-CT was used to obtain tomographic images, but these contained too much information for theoretical analysis in their original state. Thus, the images were processed by binarization and edge extraction to obtain the cross-sectional area and circumference of the rice and buckwheat husks in the tomographic plane. The cross-sectional area and circumference were approximated as the clearance between two planes, which was used to calculate the propagation constants and characteristic impedance to consider the attenuation of sound waves. The transfer matrix method was performed to obtain the transfer matrix for the entire sample, which could then be used to derive the normal incident sound absorption coefficient of the sample.

### 3.2. Image Acquisition

**Figure 8:**
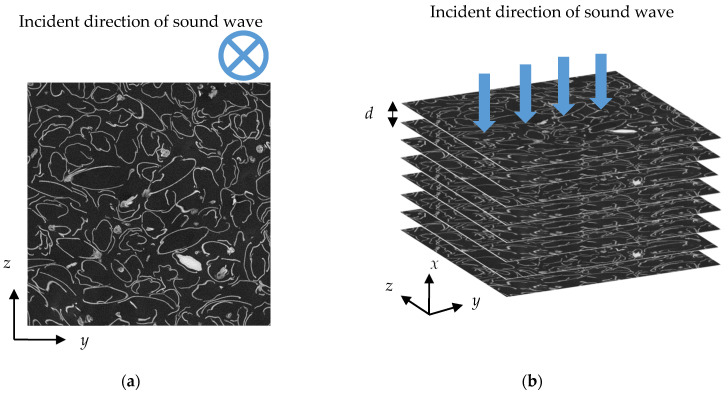
Cross-sectional image of a rice husk: (**a**) typical image with an arbitrary point in the *x*-direction; (**b**) schematic analysis.

Figure 8a shows a tomographic image of a rice husk taken by a micro-CT scan (NIKON Corp. (Tokyo, Japan) MCT225 Metrology CT). As shown in Figure 8b, the image was sliced along the *y*–*z* plane, which was perpendicular to the incident direction of the sound wave (i.e., *x*-direction). The image had dimensions of 20 mm in the *x*-direction and 25.7 mm^2^ in the *y*–*z* plane. The theoretical analysis used 884 images spanning 20 mm in the *x*-direction. The thickness *d* of an element corresponded to the pitch in the *x*-direction, which was about 22.6 µm.

### 3.3. Image Processing

#### 3.3.1. Binarization

**Figure 9:**
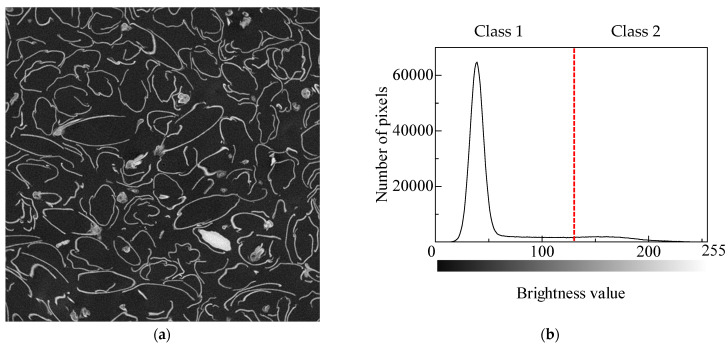
Otsu’s binarization method: (**a**) typical cross-sectional image of an arbitrary point in the *x*-direction; (**b**) the corresponding histogram (red dashed line: threshold value between class 1 and 2).

Binarization was performed to obtain the cross-sectional area of the clearance from a CT image. Binarization is an image-processing technique that converts an image with many shades of gray into a binary image with only two colors of black and white. A CT image is an 8-bit grayscale image in which each pixel can have a value of 0–255. It can be converted into a binary image by using a threshold. Otsu’s binarization [23] was used to determine the threshold, which involved finding the threshold value at which the histogram has maximum separation. As an example, Figure 9a shows a CT image of a rice husk at an arbitrary location and Figure 9b shows the corresponding histogram. The horizontal axis is the luminance, and the vertical axis is the number of pixels. Class 1 was defined as luminance values that fall on the left side of threshold value, and class 2 was defined as those that fall on the right side. The average luminance values *m*_1_ and *m*_2_ for each class are expressed by:

(2) $$m_{1}=\frac{1}{n_{1}}\sum_{i=1}^{n_{1}}x_{i}$$

(3) $$m_{2}=\frac{1}{n_{2}}\sum_{i=1}^{n_{2}}x_{i}$$

where *n*_1_ and *n*_2_ are the number of pixels in each class and *x_i_* is the luminance value of the *i*-th pixel.

Maximizing the degree of separation *σ*^2^ is synonymous with maximizing the interclass separation *σ_b_*^2^, which can be defined as follows:

(4) $${\sigma_{b}}^{2}=\frac{n_{1}n_{2}{\left(m_{1}-m_{2}\right)}^{2}}{{{(n}_{1}+n_{2})}^{2}}$$

Let *t* be the threshold value at which the degree of separation between classes is maximized. Then, the cross-sectional area of the gap *S* is expressed as:

(5) $$S={l_{p}}^{2}\times \sum_{i=1}^{t}n_{i}$$

where *l_p_* is the pixel size and *n_i_* is the number of pixels at the *i*-th threshold value.

**Figure 10:**
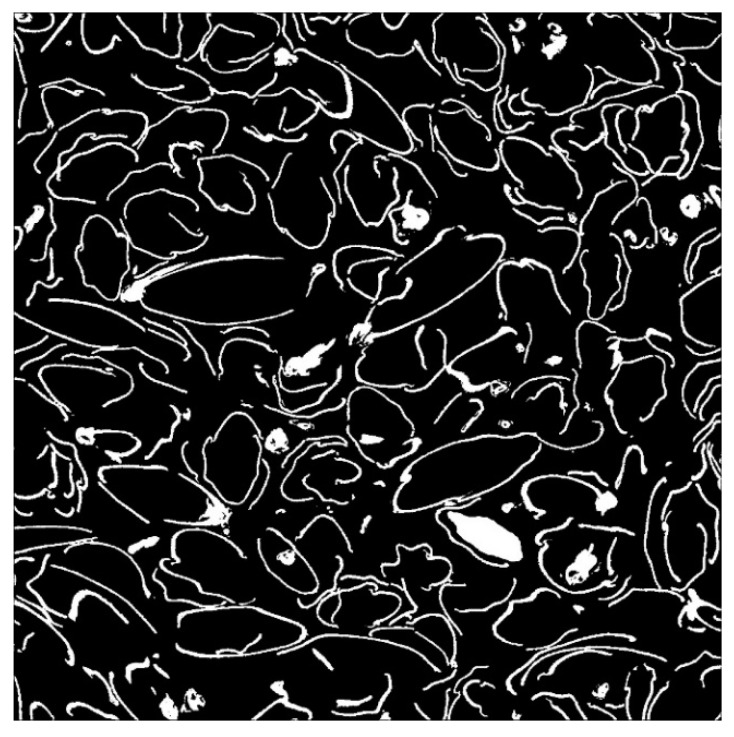
Binarized image.

Figure 10 shows a binarized image. The cross-sectional area of the gap *S* was obtained by summing the number of pixels in the area determined to be black.

#### 3.3.2. Edge Extraction

In this study, the Canny edge detection method [24] was used for edge extraction. As a preliminary step, a Gaussian filter was applied to remove noise, which involves smoothing a pixel based on the luminance values of adjacent pixels. The weighting of the luminance values decreases according to the distance of the adjacent pixel from the target pixel. The Gaussian filter is expressed as

(6) $$f(x,y,\varepsilon )=\frac{1}{2\pi \varepsilon^{2}}exp\;\left(-\frac{x^{2}-y^{2}}{2\varepsilon^{2}}\right)$$

where *ε* is the standard deviation of the two-dimensional Gaussian distribution, which in this case was set to *ε* = 5.0. This corresponded to smoothing within a radius of about 7 pixels. The intensity *G* of the luminance gradient and its connecting direction *θ* in a two-dimensional digital image can be defined from the horizontal luminance derivative *G_x_* and normal luminance derivative *G_y_* as follows:

(7) $$\left|G\right|=\sqrt{{Gx}^{2}+{Gy}^{2}}$$

(8) $$\theta ={tan}^{-1}\frac{Gy}{Gx}$$

*θ* is used to select the optimal pixel among the eight pixels tangent to the target pixel as the tangential direction of the luminance gradient, and the line segment connecting these pixels is recognized as the edge (i.e., contour) of the image. Next, the three pixels in the direction normal to the calculated edge are considered. If the intensity of the center pixel is greater than that of the pixels at both ends, that pixel is considered the maximum, and the rest of the image is deleted. In other words, because areas with large luminance gradients inevitably have a certain width at the edges of an image, the information of differential-value pixels (i.e., non-maximum areas) that are not related to the direction of edge extension are suppressed to make individual edges stand out.

Then, the hysteresis thresholding process is applied to the extracted edges to distinguish real edges from fake edges. Two thresholds are set: the upper and lower thresholds of the luminance value gradient. If the target edge is always higher than the upper threshold, it is considered a real edge. If it is lower than the lower threshold, it is deleted as a fake edge. An edge is considered a real edge if it is connected to a portion of the edge extension that is above the upper threshold. This process also removes edges with a small number of pixels based on the assumption that edges are long lines.

**Figure 11:**
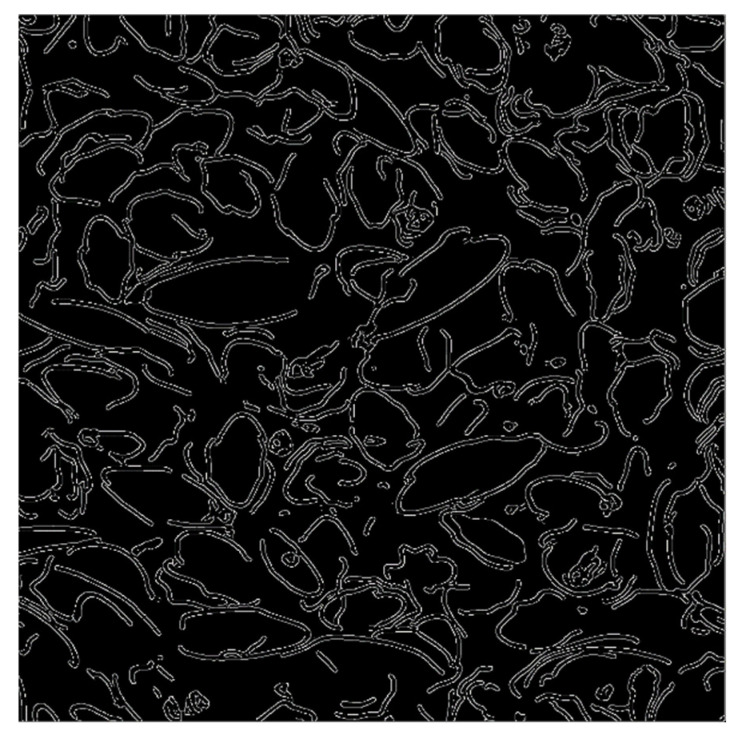
Image of rice husks with the edges extracted by the Canny edge detection method.

Figure 11 shows an example of the final image after edge extraction. Let *n_v_* be the number of edge pixels that are connected vertically or parallel to adjacent edges and *n_d_* be the number of edge pixels that are connected diagonally to adjacent edges. Then, the edge length *l* can be expressed as follows:

(9) $$l=\left(n_{v}+\left(n_{d}\times \sqrt{2}\right)\right)\times l_{p}$$

### 3.4. Derivation of the Sound Absorption Coefficient

#### 3.4.1. Approximation to Clearance between Two Planes

**Figure 12:**
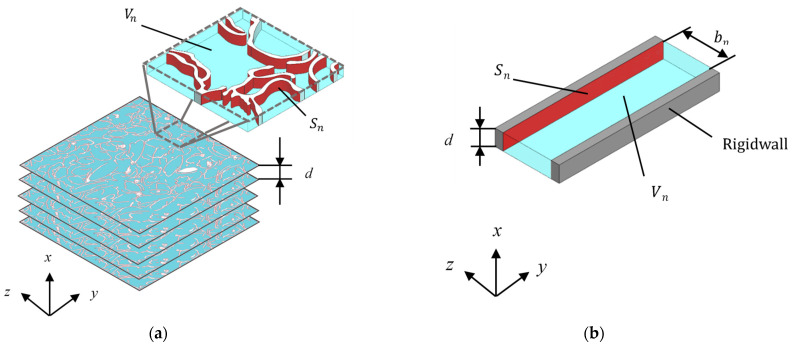
Surface area of the rice husks and volume of the clearance: (**a**) cross-sectional image of an arbitrary point in the *x*-direction; (**b**) approximation as two planes.

After the image processing, the sound absorption coefficient can be derived. The first step is to approximate the voids as the clearance between two planes. Figure 12 shows the shapes before and after approximation. Multiplying the cross-sectional area of the gap *S* obtained from image binarization (Section 3.3.1) by the pitch *d* of the image yields the volume of the gap *V_n_*, as shown in Figure 12a. Similarly, multiplying the total circumference of the cross-section obtained from edge extraction (Section 3.3.2) by *d* yields *S_n_*, as shown in Figure 12a. From this, for a single image with the pitch *d*, the gap thickness *b_n_* between the two planes shown in Figure 12b can be obtained and expressed as follows:

(10) $$b_{n}=\frac{2V_{n}}{S_{n}\times F}$$

**Figure 13:**
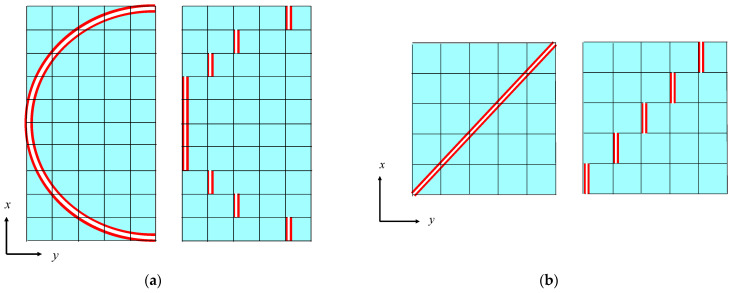
Cross-sectional view of two typical shapes: (**a**) hemispherical flake; (**b**) flat flake inclined at 45°.

*F* is a correction factor for obtaining the real surface area, and it is defined as the ratio of the real surface area to *S_n_*. As shown in the left side of Figure 13a, if a flake is assumed a hemisphere, then the flake on *x–y* plane is stepped as shown on the right side as a result of the CT scan, and *F* = π/2 ≅ 1.507. As it is also shown on the left side of Figure 13b, if a flake is assumed to have a flat plate inclined 45° in the *x*-direction, then *F* = √2 ≅ 1.414.

**Figure 14:**
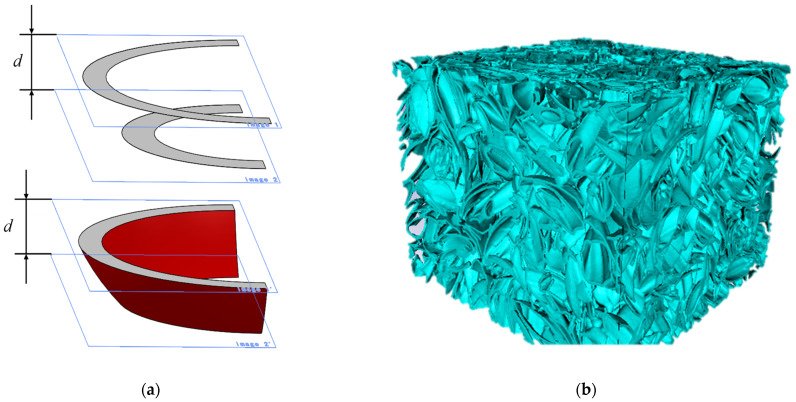
Three-dimensional model created from the CT images: (**a**) complementing the line segments with a curved surface; (**b**) 3D model of rice husks created with Simpleware.

To accurately calculate the surface area of samples, the Simpleware software (https://www.synopsys.com/company.html, accessed on 10 August 2023) was used to generate three-dimensional curved surfaces for the front and back of flakes. For the CT image shown in the upper side of Figure 14a, the front and back surfaces of the flakes were generated by complementing the line segments comprising the cross-section of each flake between adjacent images with a curved surface, as shown in the lower side of Figure 14a. Figure 14b shows an example image of a 3D model of flakes with the generated surfaces. The surface area of the 3D model was considered close to the surface area of the sample captured by the CT image. The ratio of the surface area *S_n_* to the surface area of the 3D model is expressed by the correction factor *F*. Based on the 3D model, the correction factors for rice and buckwheat husks were calculated as *F* = 1.37 and *F* = 1.43, respectively. In addition, the effects of varying the correction factor *F* from 1.0 to 1.5 in steps of 0.1 were investigated for both rice and buckwheat husks.

#### 3.4.2. Propagation Constants and Characteristic Impedance

After the clearance between two planes is approximated, the propagation constants, and characteristic impedances can be derived while considering the attenuation of sound waves. Previous studies have obtained the propagation constants and characteristic impedances considering the viscosity of air inside a tube. Tijdeman [25] and Stinson [26] considered circular tubes, Stinson and Champou [27] considered equilateral triangular tubes, and Beltman et al. [28] considered rectangular tubes. Allard [29] considered the degree of tortuosity. In this study, the methods of Stinson and Champou [27] and Allard [29] were applied.

**Figure 15:**
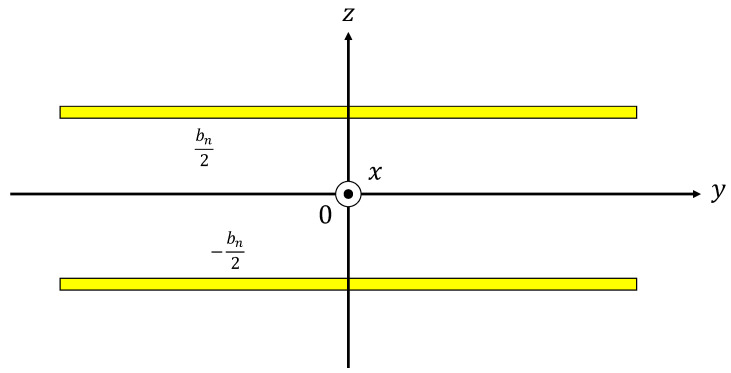
Cartesian coordinate system for the space between two parallel planes.

Figure 15 shows a Cartesian coordinate system for the space between two parallel planes, for which the effective density *ρ_s_* and compressibility *C_s_* can be derived from a three-dimensional analysis using the Navier–Stokes equations, gas equation of state, continuity equation, energy equation, and the dissipative function representing heat transfer:

(11) $$\rho_{s}=\rho_{0}{\left[1-\frac{\tanh\;\left(\sqrt{j}\lambda_{s}\right)}{\sqrt{j}\lambda_{s}}\right]}^{-1},\;\lambda_{s}=\frac{b_{n}}{2}\sqrt{\frac{\omega \rho_{0}}{\eta }}$$

(12) $$C_{s}=\left(\frac{1}{\kappa P_{0}}\right)\left\{1+\left(\kappa -1\right)\left[\frac{\tanh\;\left(\sqrt{jN_{pr}}\lambda_{s}\right)}{\sqrt{jN_{pr}}\lambda_{s}}\right]\right\}$$

where *ρ*_0_ is the density of air, *λ_s_* is the mediator variable, *b_n_* is the clearance between two planes, *ω* is the angular frequency, *η* is the viscosity of air, *κ* is the specific heat ratio of air, *P*_0_ is the atmospheric pressure, and *N_pr_* is the Prandtl number.

The propagation constant *γ* and characteristic impedance *Z_c_* can be expressed by using the effective density *ρ_s_* and compressibility *C_s_*:

(13) $$\gamma =j\omega \sqrt{\rho_{s}C_{s}}$$

(14) $${\;Z}_{c}=\sqrt{\frac{\rho_{s}}{C_{s}}}$$

By using the effective density multiplied by the tortuosity, the propagation constant and characteristic impedance considering tortuosity can be obtained [29]. Therefore, the propagation constant and characteristic impedance considering the tortuosity *α*_∞_ are expressed as follows:

(15) $$\gamma =j\omega \sqrt{\alpha_{\infty }\rho_{s}C_{s}}$$

(16) $${\;Z}_{c}=\sqrt{\frac{\alpha_{\infty }\rho_{s}}{C_{s}}}$$

#### 3.4.3. Transfer Matrix

**Figure 16:**
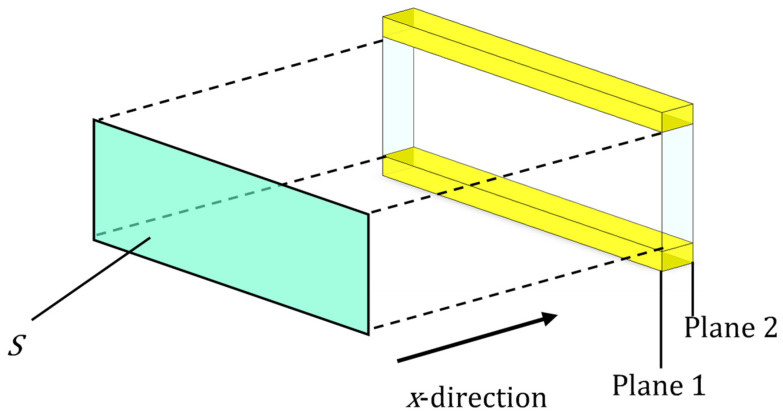
Sound incident area, incident plane, and transmission plane of the approximated clearance between two planes shown in Figure 15.

The clearance between two planes was analyzed by using the transfer matrix method for the sound pressure and volume velocity based on the one-dimensional wave equation. Figure 16 shows a schematic diagram of the clearance between two planes shown in Figure 15, which is expressed as one element in the *x*-direction. The cross-sectional area *S* of the clearance, the pitch *d* per layer, the characteristic impedance *Z_c_*, and the propagation constant *γ* can be used to obtain the transfer matrix *T* and four-terminal constants *A–D* of the acoustic tube element:

(17) $$T=\left[\begin{array}{cc} \cosh\;\left(\gamma d\right) & \frac{Z_{c}}{S}sinh\left(\gamma d\right)\\ \frac{S}{Z_{c}}sinh\left(\gamma d\right) & \cosh\;\left(\gamma d\right) \end{array}\right]=\left[\begin{array}{cc} A & B\\ C & D \end{array}\right]$$

Let the sound pressure and particle velocity in plane 1 be *p*_1_ and *u*_1_, respectively, and the sound pressure and particle velocity in plane 2 be *p*_2_ and *u*_2_, respectively. Then, the transfer matrix is expressed as follows:

(18) $$\left[\begin{array}{c} p_{1}\\ Su_{1} \end{array}\right]=\left[\begin{array}{cc} A & B\\ C & D \end{array}\right]\left[\begin{array}{c} p_{2}\\ Su_{2} \end{array}\right]$$

**Figure 17:**
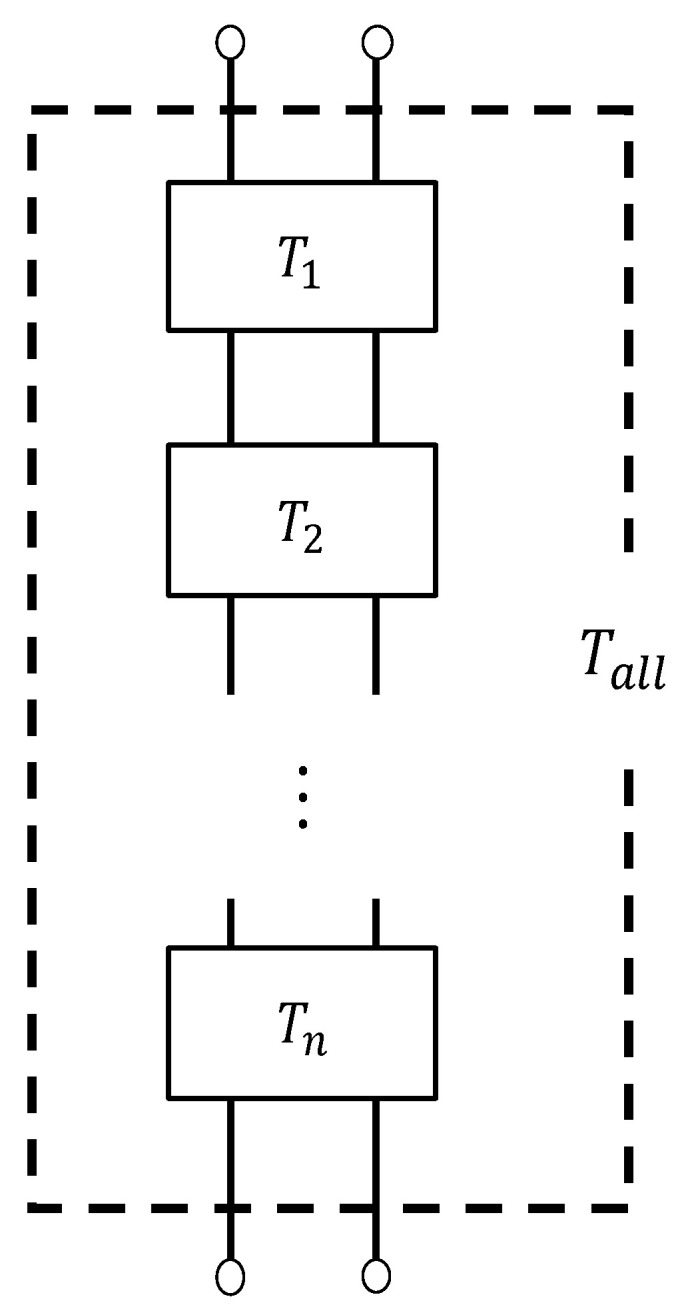
Equivalent circuit of the whole sample.

Applying Equation (18) to the gap between the two planes obtains the transfer matrix for each divided element. Because each divided element is continuous in the *x*-direction, the transfer matrix *T_all_* for the entire sample can be calculated by cascading the transfer matrices of each divided element based on the equivalent circuit shown in Figure 17. Here, *n* = 884 transfer matrices were cascaded, which is equivalent to the number of images for each sample.

#### 3.4.4. Normal Incident Sound Absorption Coefficient

The sound absorption coefficient was calculated from the transfer matrix *T_all_*. For the acoustic tube shown in Figure 16, plane 2 can be considered a rigid wall. Therefore, because the particle velocity *u*_2_ = 0, Equation (18) can be transformed as follows:

(19) $$\left[\begin{array}{c} p_{1}\\ Su_{1} \end{array}\right]=\left[\begin{array}{cc} A & B\\ C & D \end{array}\right]\left[\begin{array}{c} p_{2}\\ 0 \end{array}\right]$$

This allows us to obtain:

(20) $$\left[\begin{array}{c} p_{1}\\ Su_{1} \end{array}\right]=\left[\begin{array}{c} Ap_{2}\\ Cp_{2} \end{array}\right]$$

Let the sound pressure and particle velocity immediately outside plane 1 be *p*_0_ and *u*_0_, respectively. Then, the specific acoustic impedance *Z*_0_ from the sample’s plane of incidence in the interior can be expressed as follows:

(21) $$Z_{0}=\frac{p_{0}}{u_{0}}$$

Therefore, by *p*_0_ = *p*_1_, *S*_0_*u*_0_ = *Su*_1_, and Equation (21), the specific acoustic impedance *Z*_0_ of the sample can be expressed as follows:

(22) $$Z_{0}=\frac{p_{0}}{u_{0}}=\frac{p_{0}}{u_{0}S_{0}}S_{0}=\frac{p_{1}}{u_{1}S}S_{0}=\frac{A}{C}S_{0}$$

The relationship between the specific acoustic impedance *Z*_0_ and reflectance *R* is expressed as follows:

(23) $$R=\frac{Z_{0}-\rho_{0}c_{0}}{Z_{0}+\rho_{0}c_{0}}$$

The following relationship between the sound absorption coefficient and reflectance and Equation (23) can be used to obtain the theoretical value for the normal incident sound absorption coefficient *α* of the sample:

(24) $$\alpha =1-{\left|R\right|}^{2}$$

Thus, we present a supplemental note for the readers interested in sound insulation. Using the four-terminal constants *A*–*D* in Equation (17), the normal incident transmission loss can be determined according to a previous report [30]. However, the high porosity of the sample used in this paper does not exhibit high sound-insulation performance.

## 4. Results and Discussion

**Figure 18:**
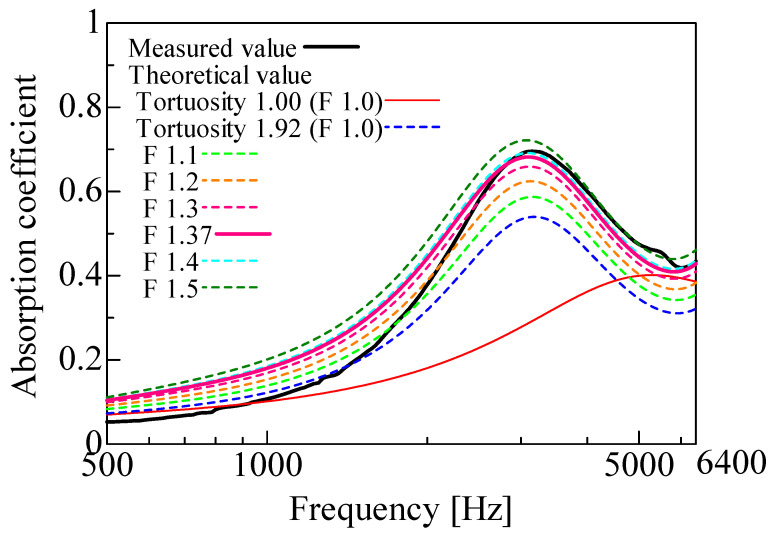
Comparison between the experiment and calculations (rice husks, *l* = 20 mm).

**Figure 19:**
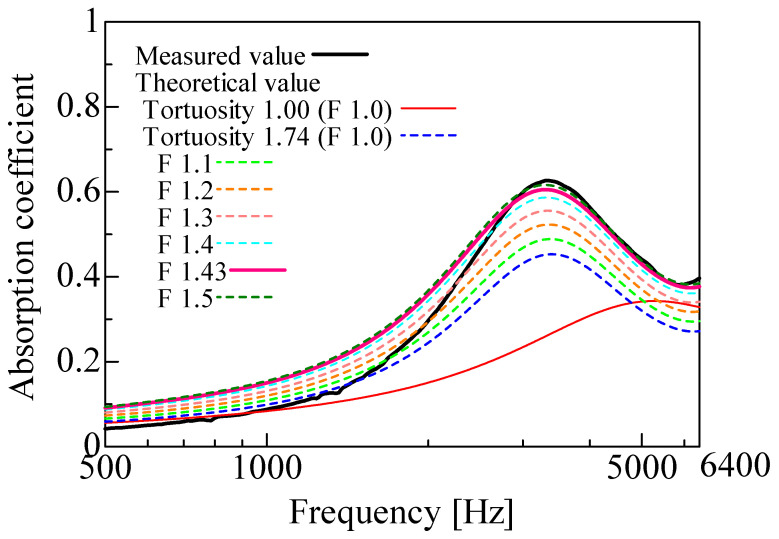
Comparison between experiment and calculations (buckwheat husks, *l* = 20 mm).

The measured and theoretical values of the normal incident sound absorption coefficient were compared for the rice husk and buckwheat husk samples. For the theoretical values, the correction factor *F* in Equation (10) was varied from 1.0 to 1.5 to evaluate its effect. The theoretical values using the correction factor *F* (shown in Table 2) to obtain the real surface area derived from the 3D model were also obtained. Figure 18 shows the results for the rice husks, and Figure 19 shows the results for the buckwheat husks. Table 3 and Table 4 present the measured and theoretical values, respectively, of the peak sound absorption frequency and peak sound absorption.

First, the measured values (black line) and theoretical values (red line) were compared without considering the tortuosity. For both the rice and buckwheat husks, the theoretical peak sound absorption frequency without considering tortuosity was higher than the measured frequency, and the theoretical peak sound absorption was lower than the measured value. When the tortuosity was considered, the peak sound absorption frequency shifted lower for both the rice and buckwheat husks, which increased the peak sound absorption. Thus, considering the tortuosity decreased the difference between the theoretical and measured values, a tortuosity greater than 1 means that the propagation path of sound waves in the sample increased in length, which has the same effect as an increase in the sample thickness. This explains the lower frequency. Based on the aforementioned findings, the peak frequency of sound absorption is related to the thickness of the layer of the sound-absorbing material, which must be thick enough to accommodate low frequencies.

In general, the sound absorption coefficient of a porous material is greatly affected by its thickness [31,32]. In other words, the sound absorption peak appears at a frequency where the thickness of the porous material corresponds to one-fourth of the wavelength of the sound wave. The peak sound absorption frequency can be decreased by a decrease in the apparent sound velocity of a material due to boundary-layer friction or tortuosity, which is equivalent to an increase in the apparent thickness of the material. In other words, increasing the tortuosity decreases the peak sound absorption frequency, which often also increases the sound absorption coefficient. For the same reason, the theoretical peak sound absorption increased when the tortuosity was considered. Overall, the accuracy of the theoretical values improved when the tortuosity was considered, and the measured tortuosity values for both the rice and buckwheat husks appear reasonable.

The theoretical values for both the rice and buckwheat husks with the correction factor *F* were close to the experimental values. The peak sound absorption increased with an increasing correction factor, which is because the attenuation of sound waves due to boundary-layer viscosity increases with a greater surface area. The theoretical values with the correction factor *F* calculated from the 3D model were close to the experimental values near the sound absorption peaks. This indicates that the theoretical surface area was closer to the actual surface area of the sample when the correction factor *F* was included. Therefore, the theoretical values with the correction factor *F* calculated from the 3D model were generally valid. Further, theoretical analyses reveal that the sound absorption characteristics rely on the geometric features of the voids; therefore, the sound absorption characteristics do not vary considerably until water is impregnated in the voids. However, the sound absorption properties may slightly vary owing to the variation in the gaps between the particles upon the inclusion of water droplets among the particles [33].

The differences between the measured and theoretical values are discussed in this section. A factor may be that the method used to estimate the attenuation of sound waves in gaps [27] has previously been shown [34] to be inaccurate for larger gaps and grain sizes. When the correction factor *F* was considered, the sound absorption coefficient was calculated to be as large as 0.05 in the low-frequency range. The sound absorption coefficient is defined as 1 minus the reflectance. Therefore, the estimation error was not large because it was 0.05 at most for a reflection coefficient of 0.8–0.9 (0.2–0.1 for the sound absorption coefficient). For both the rice and buckwheat husks, the theoretical values of the peak sound absorption frequency and peak sound absorption were close to the measured values when the tortuosity and correction factor were considered. Therefore, the theoretical values calculated by the mathematical model were considered reasonable.

## 5. Conclusions

The normal incident sound absorption coefficients of rice and buckwheat husks were calculated theoretically. The tortuosity was measured for each material and was considered in the calculation. Then, the theoretical values were compared with the measured values. The following results were obtained:

1. The structures filled with rice and buckwheat husks were not periodic, which made constructing a geometric model difficult. Therefore, the sound absorption coefficient was estimated theoretically by first processing CT images.
2. The tortuosity increased the theoretical value of the peak sound absorption and lowered the frequency, which decreased the difference with the measured values. Therefore, the measured tortuosity was considered reasonable.
3. We used a correction factor to bring the surface area of the granular material closer to the actual surface area and observed that the tortuosity obtained theoretical values that matched the trend of the measured values. These results indicate that using CT images to estimate the sound absorption coefficient is a viable approach.
4. Mass production application studies based on this research are under consideration.

## Figures and Tables

**Table 1:** Sample specifications.

Material	Average Grain Size (mm)	Mass per Grain (mg)	Bulk Density (kg/m^3^)	Measured Tortuosity
Rice husk	7.3 × 3.6	2.15	105.36	1.92
Buckwheat husk	5.7 × 4.1	4.54	110.66	1.74

**Table 2:** Surface area from the CT images and 3D model.

	Surface Area Calculated from CT Images (mm^2^)	Surface Area in the 3D Model (mm^2^)	Correction Factor *F*
Rice husk	49,383	67,481	1.37
Buckwheat husk	41,461	59,403	1.43

**Table 3:** Frequency and absorption coefficient at peak (rice husks, *l* = 20 mm).

	Peak Frequency (Hz)	Absorption Coefficient at Peak	Tortuosity	Correction Factor *F*
Measured value	3150	0.696	-	-
Theoretical value	5288	0.401	1.00	1.00
Theoretical value (Considering tortuosity)	3175	0.540	1.92	1.00
Theoretical value (Considering surface correction)	3100	0.682	1.92	1.37

**Table 4:** Frequency and absorption coefficient at peak (buckwheat husks, *l* = 20 mm).

	Peak Frequency (Hz)	Absorption Coefficient at Peak	Tortuosity	Correction Factor *F*
Measured value	3325	0.627	-	-
Theoretical value	5275	0.343	1.00	1.00
Theoretical value (Considering tortuosity)	3400	0.453	1.74	1.00
Theoretical value (Considering surface correction)	3313	0.605	1.74	1.43

## Data Availability

Data is contained within the article.
